# Supplementary material for: Causal Relationship between Aging and Anorexia Nervosa: A White-Matter-Microstructure-Mediated Mendelian Randomization Analysis
Source: Biomedicines. 2024 Aug 16;12(8):1874. doi: 10.3390/biomedicines12081874 (PMC11351342; doi:10.3390/biomedicines12081874)
Supplement: Supplementary file 1 [file biomedicines-12-01874-s001.zip › Supplementary Figure/Figure S4/Singe SNP plot of MR analysis for TL to ubm-b-1834.pdf]

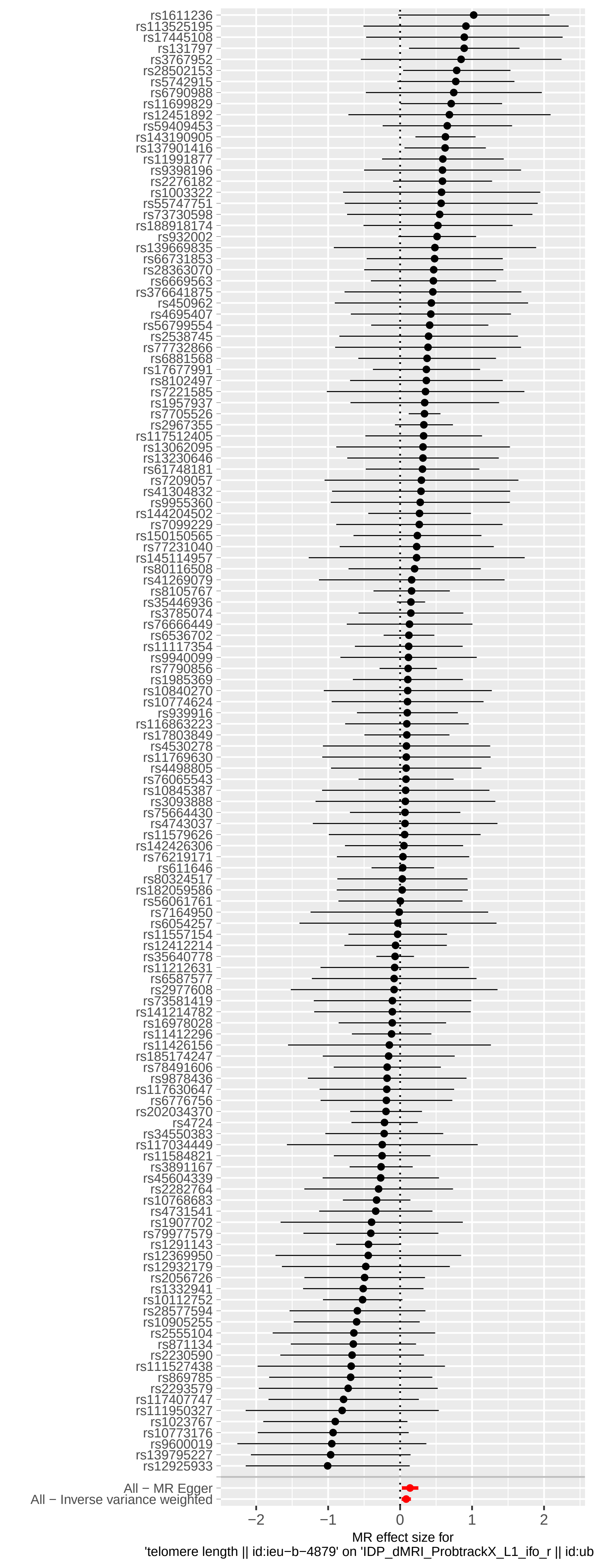

rs1611236
rs113525195
rs17445108
rs131797
rs3767952
rs28502153
rs5742915
rs6790988
rs11699829
rs12451892
rs59409453
rs143190905
rs137901416
rs11991877
rs9398196
rs2276182
rs1003322
rs55747751
rs73730598
rs188918174
rs932002
rs139669835
rs66731853
rs28363070
rs6669563
rs376641875
rs450962
rs4695407
rs56799554
rs2538745
rs77732866
rs6881568
rs17677991
rs8102497
rs7221585
rs1957937
rs7705526
rs2967355
rs117512405
rs13062095
rs13230646
rs61748181
rs7209057
rs41304832
rs9955360
rs144204502
rs7099229
rs150150565
rs77231040
rs145114957
rs80116508
rs41269079
rs8105767
rs35446936
rs3785074
rs76666449
rs6536702
rs11117354
rs9940099
rs7790856
rs1985369
rs10840270
rs10774624
rs939916
rs116863223
rs17803849
rs4530278
rs11769630
rs4498805
rs76065543
rs10845387
rs3093888
rs75664430
rs4743037
rs11579626
rs142426306
rs76219171
rs611646
rs80324517
rs182059586
rs56061761
rs7164950
rs6054257
rs11557154
rs12412214
rs35640778
rs11212631
rs6587577
rs2977608
rs73581419
rs141214782
rs16978028
rs11412296
rs11426156
rs185174247
rs78491606
rs9878436
rs117630647
rs6776756
rs202034370
rs4724
rs34550383
rs117034449
rs11584821
rs3891167
rs45604339
rs2282764
rs10768683
rs4731541
rs1907702
rs79977579
rs1291143
rs12369950
rs12932179
rs2056726
rs1332941
rs10112752
rs28577594
rs10905255
rs2555104
rs871134
rs2230590
rs111527438
rs869785
rs2293579
rs117407747
rs111950327
rs1023767
rs10773176
rs9600019
rs139795227
rs12925933
All − MR Egger
All − Inverse variance weighted
−2
−1
0
1
2
MR effect size for
'telomere length || id:ieu−b−4879' on 'IDP_dMRI_ProbtrackX_L1_ifo_r || id:ub
